# Contrast-Enhanced Harmonic Endoscopic Ultrasound-Guided Fine-Needle Aspiration in the Diagnosis of Solid Pancreatic Lesions: A Retrospective Study

**DOI:** 10.1371/journal.pone.0121236

**Published:** 2015-03-20

**Authors:** Xiaojia Hou, Zhendong Jin, Can Xu, Minmin Zhang, Jianwei Zhu, Fei Jiang, Zhaoshen Li

**Affiliations:** Department of Gastroenterology, Changhai Hospital, Second Military Medical University, Shanghai, 200433, China; University of Utah School of Medicine, UNITED STATES

## Abstract

**Background:**

The negative predictive value of endoscopic ultrasonography-guided fine needle aspiration for the diagnosis of solid pancreatic lesions remains low, and the biopsy specimens are sometimes inadequate for appropriate pathological diagnosis.

**Aims:**

To evaluate the usefulness of a novel method of contrast-enhanced harmonic endoscopic ultrasonography-guided fine-needle aspiration for the differential diagnosis and adequate sampling of solid pancreatic lesions.

**Methods:**

Patients with a diagnosis of solid pancreatic lesions who underwent fine-needle aspiration guided by contrast-enhanced harmonic endoscopic ultrasonography or by endoscopic ultrasonography from October 2010 to July 2013 were retrospectively identified and classified into the CH-EUS or EUS group, respectively. Surgical pathology and/or follow-up results were defined as the final diagnosis. Operating characteristics and adequacy of biopsy specimens by fine-needle aspiration were compared between the two groups.

**Results:**

Operating characteristics for contrast-enhanced harmonic endoscopic ultrasonography-guided fine-needle aspiration in solid pancreatic lesions were as follows: area under the curve = 0.908, sensitivity = 81.6%, specificity = 100%, positive predictive value = 100%, negative predictive value = 74.1%, and accuracy = 87.9%. The percentage of adequate biopsy specimens in the CH-EUS group (96.6%) was greater than that in the EUS group (86.7%).

**Conclusion:**

Simultaneous contrast-enhanced harmonic endoscopic ultrasonography during fine-needle aspiration is useful for improving the diagnostic yield and adequate sampling of solid pancreatic lesions.

## Introduction

The differential diagnosis of solid pancreatic lesions is a common clinical challenge. In this context, the therapeutic decision is mainly based on the ability to establish or exclude malignancy [1]. Endoscopic ultrasonography (EUS) is the most sensitive imaging procedure available for characterizing pancreatic tumors. EUS is effective in identifying smaller sized tumors [1–3], especially those smaller than 2 cm [2,4]. The sensitivity of conventional EUS for detecting pancreatic adenocarcinomas is 89–100% [5–8]. Although EUS has a high overall sensitivity, it remains difficult to differentiate pancreatic cancer from other solid lesions on the basis of only endosonographic features [2,9–14].

EUS-guided fine needle aspiration (EUS-FNA) is a safe and useful method for pancreatic tissue sampling [13–15]. However, the biopsy specimens of EUS-FNA are sometimes inadequate for appropriate pathological diagnosis because of possible sampling errors [16–17]. Due to the potential for false-negative (FN) and nondiagnostic results, selecting an appropriate treatment strategy is challenging for patients with inconclusive EUS-FNA diagnoses. A recent meta-analysis reported that having an on-site cytopathologist can enhance the accuracy and efficiency of EUS-FNA [18], but some centers do not have this capability. Hence, it is necessary to improve the diagnostic yield and sampling adequacy of EUS-FNA.

Contrast-enhanced EUS (CE-EUS), which indicates vascularization in pancreatic lesions, has been found to be useful in the differential diagnosis of pancreatic tumors, especially those with small sizes [19,20]. However, CE-EUS has several limitations, such as blooming artifacts, poor spatial resolution, and low sensitivity to slow flow [21,22]. To overcome these limitations, contrast-enhanced harmonic endoscopic ultrasonography (CH-EUS) with a second-generation ultrasound contrast agent was recently developed. This technology can detect signals from microbubbles in vessels with a very slow flow without Doppler-related artifacts, can be used to characterize microvascularity in the pancreas, and can aid in the differentiation of solid pancreatic masses [21,22]. Some groups reported that CH-EUS was very sensitive and accurate (sensitivity = 89–96%; accuracy = 82–95%) with a high negative predictive value (NPV = 88–93%) in the diagnosis of pancreatic adenocarcinoma as hypovascular masses [19,21–25]. Moreover, CH-EUS allows visualization of the microvasculature, resulting in detailed observation of the intratumoral structure and characterization of difficult cases [22].

Because CH-EUS reveals not only parenchymal perfusion but also the microvasculature of the pancreas, it can be used to differentiate pancreatic neoplasia from other pancreatic diseases [12,26,27]. Compared to conventional EUS, CH-EUS can improve the observation of pancreatic tumors and help identify different pathological areas of pancreatic lesions. However, there are few studies of CH-EUS-guided fine-needle aspiration (CH-EUS-FNA) for the diagnosis and adequate sampling of pancreatic masses [9]. The aim of this study was to evaluate the usefulness of a novel method of fine-needle aspiration (FNA) guided by simultaneous CH-EUS for the differential diagnosis and adequate sampling of solid pancreatic lesions.

## Materials and Methods

### Study design and population

This was a retrospective, case-control study. Patients with a diagnosis of solid pancreatic lesions based on imaging (CT scan and/or MRI) who underwent EUS-FNA or CH-EUS-FNA from October 2010 to July 2013 were retrospectively identified from a prospectively collected endoscopy database at our center. Patients were classified into the CH-EUS or EUS group. Only patients with surgical pathology or with at least 12 months of clinical follow-up after EUS-FNA or CH-EUS-FNA were included in this study. Two authors (X.H. and J.Z.) reviewed the computerized patient record system to obtain patient demographics, lesion sites, EUS or CH-EUS characteristics of the lesion, and clinical follow-up information.

The study protocol conformed to the guidelines of the 1975 Declaration of Helsinki (6th revision, 2008) and was approved by the Institutional Review Board of Changhai Hospital, Second Military Medical University. Written informed consent was obtained from all patients before undergoing EUS-FNA or CH-EUS-FNA. Patients described in this manuscript gave written informed consent (as outlined in the PLOS consent form) to publish their case details.

### EUS-FNA and CH-EUS-FNA technique

Procedures were performed on each patient by an experienced endosonographer (Z.J.) under intravenous propofol anesthesia, as described previously [28]. A radial endoscopic ultrasound (GFUCT2000, Olympus, Japan) was used to characterize the lesion. In the CH-EUS group, a linear echoendoscope (GFUC-30P, Olympus, Japan) and a processor (Prosound Alpha 10, Aloka, Tokyo, Japan) were used to perform CH-EUS-FNA with a mechanical index of 0.4. Lesions were imaged in real-time with the extended pure harmonic detection (ExPHD) mode, which is specific for CH-EUS, and simultaneous monitoring by the fundamental B mode. An intravenous injection of 4.8 mL of SonoVue (Bracco, Milan, Italy) was administered through an antecubital vein with a 20-G catheter, followed by a 20-mL saline flush. Pancreatic lesion enhancement was compared with the adjacent pancreatic parenchyma, and three patterns were differentiated as hypo-, iso-, or hyperenhancement [9].

Two other blinded readers (C.X., M.Z.) made independent readings on-site and quickly reassessed any discrepant findings together with the operator to reach agreement regarding the CH-EUS diagnosis. When a pancreatic lesion was diagnosed as pancreatic adenocarcinoma by CH-EUS, the hypoenhancement area of the lesion was selected as an aspiration site. When the CH-EUS diagnosis was focal pancreatitis or neuroendocrine tumor, the iso- or hyperenhancement area of the lesion was selected, according to previous studies [9,12]. The biopsy site was monitored simultaneously by endosonography. Patients with solid masses in the pancreas head and an uncinate process of the pancreas underwent biopsies via a transduodenal approach. Masses in the neck, body, or tail of the pancreas were targeted via a transgastric approach. Lesions were sampled by a 22-gauge needle (Wilson Cook Medical Inc., Winston Salem, NC, USA) under CH-EUS guidance.

In the EUS group, color Doppler was used to exclude any vessel in the path of the needle before the lesions were sampled under EUS guidance. A mass was punctured a minimum of three times with the same needle.

Needle aspirate was placed on glass slides. Ethanol-fixed smears were prepared, stained with Papanicolaou stain, and evaluated the next working day by a cytopathologist to make the preliminary diagnosis. Any residual aspirate was collected into a liquid preservative for subsequent preparation of a ThinPrep slide and a cell block. Immunocytochemistry was performed on the cell block preparation when indicated. No cytopathologist was present in the endoscopy room for the on-site sample evaluation.

### Cytologic interpretation

Cytologic diagnoses were categorized into the following groups [29]: malignancy (M), suspicious for malignancy (S), atypical-indeterminate for malignancy (A), benign (B), or nondiagnostic (ND). Aspirate smears with scant cellularity were defined as nondiagnostic. The preliminary cytologic diagnosis was reviewed by two cytopathologists with the same criteria in a blinded fashion. Thereafter, the two observers reassessed any discrepant findings together to reach an agreement as to the final cytologic diagnosis.

### Final diagnosis

The final diagnosis of pancreatic malignancy was based on a gold standard [30]. Either of the following was sufficient for a diagnosis of malignancy: (i) surgical pathology of malignancy, as reviewed by two expert pathologists; or (ii) unequivocally malignant cytology with clinical progression compatible with the diagnosis, or death from malignancy. Patients who had a benign mass by FNA that was not confirmed by surgical pathology were monitored for at least 12 months. Absence of disease progression or resolution of the imaging or clinical changes was required for the diagnosis of benign disease.

### Statistical analysis

Statistical analyses were performed with the SPSS (version 18.0; IBM, NY, USA) and MedCalc software packages (version 12.7.7; MedCalc Software bvba, Ostend, Belgium). Continuous variables are presented as medians and ranges of values. Categorical variables are reported as proportions with 95% confidence intervals (CIs) where appropriate. Categorized variables were compared by the Fisher’s exact or Chi-squared two-tailed test, as appropriate. Quantitative variables were analyzed by the two-sample Student *t*-test (for normal distributions) or the Mann-Whitney U-test (for skewed distributions).

Receiver operating characteristic (ROC) analysis was performed to estimate the diagnostic accuracy of CH-EUS-FNA and EUS-FNA in pancreatic malignancy. The area under the ROC curve (AUC), sensitivity, specificity, positive predictive value (PPV), NPV, and accuracy were calculated with 95% CIs. Two independent AUCs were compared by the Z-test. Cases of malignancy were considered positive. Patients with cytologic diagnoses of nondiagnostic, benign, atypical, or suspicious were considered FN cases if the final diagnosis was malignancy. The percentage of adequate samples, cost-effectiveness, and incremental cost for per diagnostic sample were analyzed. *P* < 0.05 was considered statistically significant.

## Results

### Patient characteristics

A total of 163 patients were eligible for the study from October 2010 to July 2013. No complications were observed in any of the cases. Final diagnoses in the CH-EUS group (*n* = 58) were pancreatic adenocarcinoma (35 cases, 60%), focal pancreatitis (20 cases, 34%), and neuroendocrine tumor (3 cases, 6%). Final diagnoses in the EUS group (*n* = 105) were pancreatic adenocarcinoma (65 cases, 62%), focal pancreatitis (33 cases, 31%), and neuroendocrine tumor (7 cases, 7%).

Baseline characteristics of the patients are summarized in Table 1. There was no significant difference between the two groups with respect to age, gender, location or size of the mass, number of passes, or final diagnosis. Of the 100 patients who were finally diagnosed with pancreatic adenocarcinoma, 35 cases (35%) of disease were confirmed by histological evaluation of surgically resected tissue specimens, and 65 cases (65%) were confirmed by clinical progression or death from malignancy within 3 to 18 months. All 53 patients with a final diagnosis of benign cytology had a clinical course compatible with benign disease without progressive imaging, and all survived during a mean 13-month follow-up.

**Table 1:** Characteristics of patients, tumors, and fine-needle aspiration in the two groups (*n* = 163).

Characteristics	CH-EUS Group (*n* = 58)	EUS Group (*n* = 105)	*P*-value
Male (%)	36 (62)	63(60)	*P* = 0.80
Age (years)	55.1 ± 11.7	56.2 ± 12.5	*P* = 0.72
Tumor location, *n* (%)			*P* = 0.95
Head	35 (60)	65 (62)	
Body	14 (24)	23 (22)	
Tail	9 (16)	17 (16)	
Tumor size (cm)	3.8 ± 1.2	3.9 ± 0.8	*P* = 0.43
Number of passes (*n*)	3.7± 0.9	3.6 ± 0.8	*P* = 0.52
Final diagnosis, *n* (%)			*P* = 0.88
Adenocarcinoma	35 (60)	65(62)	
Neuroendocrine tumor	3 (6)	7 (7)	
Focal pancreatitis	20 (34)	33 (31)	

CH-EUS, contrast-enhanced harmonic endoscopic ultrasonography; EUS, endoscopic ultrasonography.

### Cytopathologic diagnosis

FNA cytopathologic diagnoses of the solid pancreatic lesions in CH-EUS group and EUS group are shown in Table 2. In the CH-EUS group, all cytopathologic diagnosis of M, S, and A were clinically or pathologically confirmed to be malignant. In the EUS group, one of the cytopathologic diagnoses of A was clinically confirmed to be focal pancreatitis. Of the cases with a benign diagnosis, 2/21 and 6/32 cases were finally identified as malignant in the CH-EUS and EUS groups, respectively. Of the 2 nondiagnostic cases in the CH-EUS group, 1 case was finally identified as malignant, and 1 case was determined to be benign. Of the 14 nondiagnostic cases in the EUS group, 8 cases were finally diagnosed as malignant and 6 cases were diagnosed as benign (Table 2).

**Table 2:** Final cytological diagnosis of solid pancreatic lesions in the two groups (*n* = 163).

Cytological diagnosis	Final diagnosis			
	CH-EUS Group		EUS Group	
	Benign	Malignant	Benign	Malignant
Malignant	0	31	0	51
Suspicious	0	3	0	5
Atypical	0	1	1	2
Benign	19	2	26	6
Nondiagnostic	1	1	6	8
Total	20	38	33	72

CH-EUS, contrast-enhanced harmonic endoscopic ultrasonography; EUS, endoscopic ultrasonography.

The FN rate in the CH-EUS group (18.4%; 95% CI, 6.1–30.7%) was lower than in the EUS group (29.2%; 95% CI, 18.7–39.7%), but the difference was not statistically significant (χ^2^ = 1.514, *P* = 0.219). The percentage of adequate biopsy specimens in the CH-EUS group (96.6%; 95% CI, 91.1–100.0%) was greater than the percentage in the EUS group (86.7%; 95% CI, 80.2–93.2%), but the increment was not statistically significant (*P* = 0.054).

### Performance characteristics

Table 3 reports the operating characteristics for CH-EUS-FNA and EUS-FNA in diagnosing malignant lesions. Operating characteristics for CH-EUS-FNA were as follows: sensitivity = 81.6% (95% CI, 65.7–92.3%), specificity = 100% (95% CI, 83.2–100.0%), PPV = 100% (95% CI, 88.8–100.0%), NPV = 74.1% (95% CI, 53.7–88.9%), accuracy = 87.9% (95% CI, 79.5–96.3%), and AUC 0.908 (95% CI, 0.803–0.969). Operating characteristics for EUS-FNA were as follows: sensitivity = 70.8% (95% CI, 58.9–81.0%), specificity = 100% (95% CI, 89.4–100%), PPV = 100.0% (95% CI, 93.0–100.0%), NPV = 61.1% (95% CI, 46.9–74.2%), accuracy = 80.0% (95% CI, 72.3–87.7%), and AUC = 0.854 (95% CI, 0.772–0.915). CH-EUS-FNA and EUS-FNA did not differ significantly in terms of diagnostic accuracy (Z = 1.030, *P* = 0.303). However, the AUC, sensitivity, NPV, and accuracy of CH-EUS-FNA were increased by 0.054, 10.8%, 13.0%, and 7.9%, respectively, compared to EUS-FNA. The distribution of operation dates was not statistically significant, and the performance characteristics were similar between the first and second half of cases in each group.

**Table 3:** Operating characteristics in diagnosing pancreatic solid malignant lesions in the two groups (*n* = 163).

	Sensitivity,% (95% CI)	Specificity,% (95% CI)	PPV,% (95% CI)	NPV,% (95% CI)	Accuracy (95% CI)	AUC (95% CI)
CH-EUS Group (*n* = 58)	81.6 (65.7–92.3)	100.0 (83.2–100.0)	100.0 (88.8–100.0)	74.1 (53.7–88.9)	87.9 (79.5–96.3)	0.908 (0.803–0.969)
CH-EUS Group* (*n* = 58)	89.5 (75.2–97.1)	100.0 (83.2–100.0)	100.0 (89.7–100.0)	83.3 (62.6–95.3)	93.1 (86.6–99.6)	0.947 (0.855–0.989)
CH-EUS Group# (*n* = 58)	92.1 (78.6–98.3)	100.0 (83.2–100.0)	100.0 (90.0–100.0)	87.0 (66.4–97.2)	94.8 (89.1–100.0)	0.961 (0.874–0.994)
EUS Group (*n* = 105)	70.8 (58.9–81.0)	100.0 (89.4–100.0)	100.0 (93.0–100.0)	61.1 (46.9–74.2)	80.0 (72.3–87.7)	0.854 (0.772–0.915)
EUS Group* (*n* = 105)	77.8 (66.4–86.7)	100.0 (89.4–100.0)	100.0 (93.6–100.0)	67.4 (52.5–80.1)	84.8 (77.9–91.7)	0.889 (0.813–0.942)
EUS Group# (*n* = 105)	80.6 (69.5–88.9)	96.9 (84.2–99.9)	98.3 (90.9–100.0)	69.6 (54.2–82.3)	85.7 (79.0–100.0)	0.888 (0.811–0.941)

CH-EUS, contrast-enhanced harmonic endoscopic ultrasonography; EUS, endoscopic ultrasonography group; AUC, area under receiver operating characteristic curve; PPV, positive predictive value; NPV, negative predictive value; 95% CI, 95% confidence interval.

*Suspicious cytology considered as diagnostic of malignancy.

#Atypical/suspicious cytology considered as a diagnostic of malignancy.

To compare the performance characteristics of the different diagnostic classes, we recalculated these values and classified suspicious/atypical cytology cases as positive results. Performance characteristics based on this reclassification are outlined in Table 3.

### Cost-effectiveness analysis

Although CH-EUS-FNA was slightly more expensive than EUS-FNA (7600 vs. 7000 CNY in China), the cost of CH-EUS-FNA per diagnostic sample (7867 CNY) was lower than that of EUS-FNA (8073 CNY). The incremental cost per diagnostic sample, defined as [(Cost of CH-EUS—Cost of EUS)/(Diagnostic rate of CH-EUS—Diagnostic rate of EUS)] was 6060 CNY, which was less than the cost of an EUS-FNA.

## Discussion

**Fig 1:**
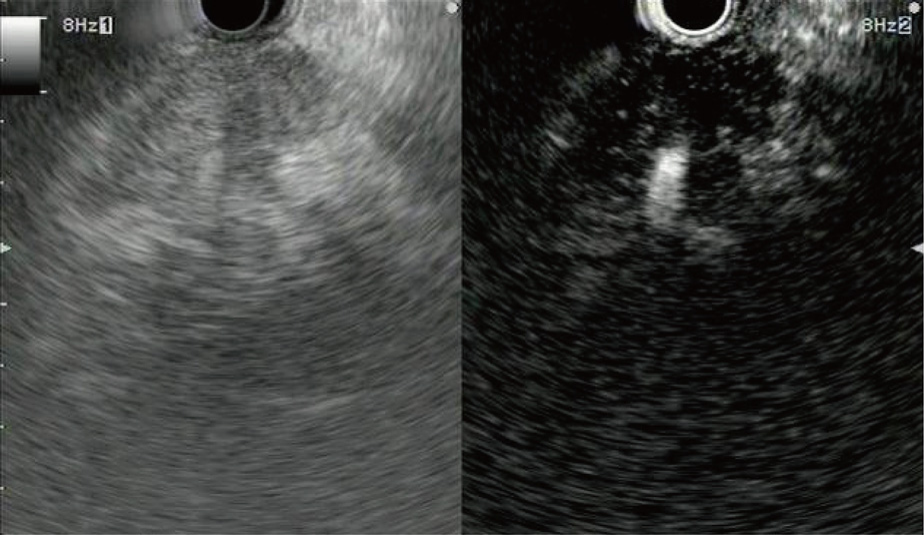
Representative example of a pancreatic adenocarcinoma with hypoenhancement. Conventional endoscopic ultrasonography (left) shows a heterogeneous hypoechoic area without a clear margin at the pancreas head. Contrast-enhanced harmonic endoscopic ultrasonography (right) indicates that most of the area is hypovascular and the remaining area is hypervascular compared to the surrounding tissue. An irregular margin is visible.

**Fig 2:**
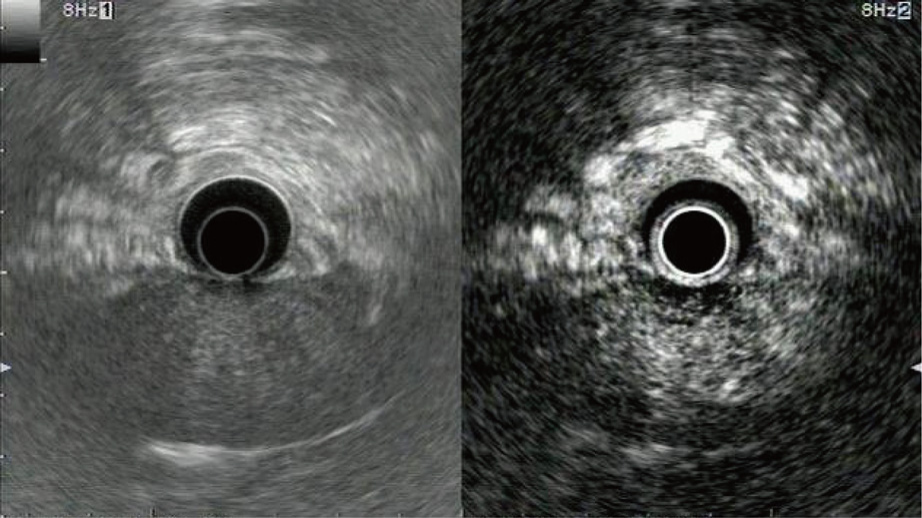
Representative example of focal pancreatitis with hyperenhancement. Conventional endoscopic ultrasonography (left) shows a slightly hypoechoic area without a clear margin at the pancreas head. Contrast-enhanced harmonic endoscopic ultrasonography (right) indicates that enhancement in this area is higher than in the surrounding tissue, and a margin is clearly visible.

**Fig 3:**
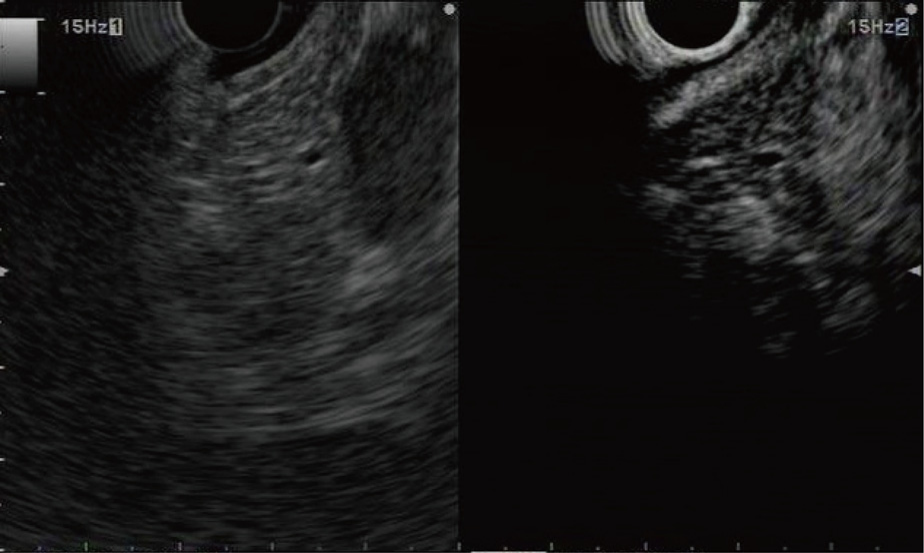
Representative example of a pancreatic neuroendocrine tumor with hyperenhancement. Conventional endoscopic ultrasonography (left) shows a hypoechoic mass with a clear margin at the pancreas body. Contrast-enhanced harmonic endoscopic ultrasonography (right) indicates that the mass has a hyperenhancement compared to the surrounding tissue.

The results of this study demonstrate that simultaneous use of CH-EUS during FNA is useful for improving the diagnostic yield and adequate sampling of solid pancreatic lesions. In contrast with EUS-FNA, CH-EUS-FNA is beneficial because it helps identify the target and pathological features of pancreatic lesions Figs. (1–3).

In a recent multicenter study of 1075 patients with solid pancreatic masses, EUS-FNA confirmed malignancy in 71% of cases and yielded suspicious or atypical cells in 11% of cases [31]. The current study compares favorably with these data. In the CH-EUS and EUS groups, 81.6% and 70.8% of cases, respectively, were confirmed to be malignant, and 10.5% and 9.7% of cases, respectively, were diagnosed as atypical/suspicious. Excepting one benign case, all of the suspicious and atypical cases were subsequently confirmed to be malignant.

Benign tissue or scant cellularity was obtained in the 7 and 21 FN diagnoses in the CH-EUS group and EUS group, respectively. These samples may have been blood samples, or there may have been underlying chronic pancreatitis or necrosis in the tumor interior. Reclassification of specimens with a suspicious or atypical cytology as malignant resulted in a FN rate of 7.9% (compared to 18.4%) in the CH-EUS group and 19.4% (compared to 29.2%) in the EUS group. These FN rates are higher than the FN rate reported in a recent meta-analysis [32]. Much of this difference may be attributed to the absent of on-site cytology [17]. The high rate of malignancy in lesions with atypical/suspicious cytology supports the performance of repeat EUS-FNA examination in these cases, or possibly treating these cytologic results as positive when there is high clinical suspicion of malignancy.

The AUC, sensitivity, NPV, and accuracy of CH-EUS-FNA for solid pancreatic masses were higher than those for EUS-FNA, but the difference was not statistically significant. The lack of significance may due to the small number of patients. Sensitivity values for pancreatic malignant lesions in both groups were lower than values reported in some previous studies [32]. The difference may be due to differences in the diagnostic interpretations of EUS-FNA cytology. For example, some studies excluded nondiagnostic specimens and/or classified specimens with an atypical/suspicious cytology as positive, which would increase the performance [14,29,33–35]. Our study produced a lower sensitivity because of the strict diagnostic cytologic criteria. When we considered atypical and suspicious reports as diagnostic of malignancy, the AUC, sensitivity, NPV, and accuracy in both groups increased.

The ability to confirm a pathologic diagnosis in both malignant and benign lesions relies heavily on obtaining adequate cellular material. In a previous study, all FN cases of EUS-FNA cytology were suggested to be due to sampling errors rather than interpretation errors [29]. Some groups reported that technical failures resulted in unsatisfactory aspirates in 1.5–13% of cases [16,17,36–41]. Other studies showed high cytologic diagnostic yields with 3 to 6 needle passes through the lesion and showed that having a cytopathologist on-site enhanced the accuracy and efficiency of EUS-FNA [13,18,37,39,42,43]. Despite recommendations for on-site cytopathology, some centers do not have this capability because of the cost and a lack of resources.

Fusaroli et al. [23] reported that CH-EUS improves the observation of pancreatic tumors, compared with conventional EUS. Conventional EUS sometimes fails to depict pancreatic tumors in cases with chronic pancreatitis, diffusely infiltrating carcinoma, or a recent episode of acute pancreatitis [44]. In cases where the target of EUS-FNA cannot be identified, the accurate insertion of the needle is hampered, and the resultant EUS-FNA aspirant contains insufficient tumor material [45]. Because CH-EUS is more sensitive, it can be used to identify the target of EUS-FNA [23–25]. CH-EUS may also help avoid puncturing necrotic and inflammatory areas of malignant masses or hard and scirrhous areas of inflammatory masses, reducing the need for repeat FNA.

Without on-site cytology, we were able to obtain an adequate sample for cytological evaluation in 96.6% of cases by CH-EUS-FNA with a mean number of 3.7 ± 0.9 passes. Although the difference was not quite statistically significant due to our relatively small sample size, the percentage of adequate biopsy specimens for CH-EUS-FNA in solid pancreatic masses was greater than the percentage for EUS-FNA. CH-EUS-FNA has a longer operating time and is slightly more expensive than EUS-FNA; however, the overall and incremental costs of CH-EUS-FNA per diagnostic sample were less than the corresponding costs of EUS-FNA. Thus, we can consider CH-EUS-FNA for pancreatic solid lesions as cost-effective.

To ascertain whether the operator experience may have contributed to bias with respect to outcomes, we compared the operation dates and performance characteristics between the first and second half of cases. The distribution of operation dates was not statistically significant, and the performance characteristics were similar in each group. Thus, improved experience is unlikely to have biased the results.

However, the current study has a few limitations: our study was a retrospective, single-center study and the number of enrolled patients did not enable us to form a definitive conclusion. Large prospective studies are required to confirm our findings. Secondly, we did not assess the number of passes, and its impact on the results cannot be excluded. Thirdly, FNA was performed by a single endoscopist without on-site cytopathology. As a result, these results might differ from those at other centers. Despite this limitation, considering the higher performance characteristics and greater percentage of adequate biopsy samples that we found, the assessment of pancreatic tumor vascularity by CH-EUS could improve the results of EUS-FNA by targeting the best area for sampling.

In conclusion, CH-EUS is a new technique that can visualize both the parenchymal perfusion and microvasculature of the pancreas without artifacts. Simultaneous CH-EUS during FNA has good performance characteristics and cost-effectiveness. It is useful for adequate sampling because it helps to identify the target for EUS-FNA and to predict the pathological features of pancreatic lesions. The combination of CH-EUS and FNA should be considered for the evaluation of solid pancreatic masses, especially some difficult FNA cases.
